# Letter from Dr. Vernois

**Published:** 1839-07-20

**Authors:** 


### LETTER FROM DR. VERNOIS.—No. I.

[During the absence, for a few months, of Professor Martins, upon a scientific expedition in the North of Europe, his place in our correspondence is supplied by Dr. Vernois, of Paris. Our readers will find that their interests have not been neglected in the substitution. Eds.]

*The Late Discussion upon the Subject of Poisoning from Arsenious Acid.*

Paris, *May 15th*, 1839.

To the Editors of the Medical Examiner.

In the absence of more important medical news, I propose to draw the attention of your readers to a question of considerable clinical and therapeutic interest, the solution of which is still under the consideration of a commission appointed by the Academy of Medicine. I allude to the subject of poisoning by arsenious acid, including some of the symptoms to which it gives rise, and the therapeutic efficacy of certain methods of treatment. My intention, you may perceive, is not to undertake the medico-legal history of the subject, but to attempt to throw a little light upon some of the points which have been started in the discussion. Two recent facts have awakened the attention of physicians to the topic. The first, noticed by all the journals of the metropolis, is the suicide, by arsenic, of Soufflard, a prisoner condemned to capital punishment; the second, an accident of a similar character, which happened to a patient of Dr. Coquerel. An account of the first case has been drawn up and published by Mr. James, interne at the Hôtel Dieu; nothing is known of the second, except some details furnished by MM. Coquerel and Orfila, followed by some notices upon the subject, contradictory of the preceding, by M. Rognetta. We necessarily encounter here doubt and hesitancy; for we cannot enter into the personal discussion which has taken place on the occasion. The principal circumstances involved in the affair, are as follows:—On the 27th of January last, a man was poisoned by arsenious acid, mixed by mistake, instead of flour, in a ragout. Continued vomiting, which lasted near forty-eight hours, was the first symptom observed. Dr. Coquerel administered the sesquioxide of iron, and had some leeches applied. When seen the fifth day by M. Orfila, the patient laboured under violent, and even painful palpitations of the heart, with symptoms of meningitis, fever, &c. A bleeding was practised, and the presence of arsenic was ascertained in the blood. On the eleventh day, the poison was again recognised in blood taken by a second bleeding. The symptoms became gradually alleviated, and M. Orfila took the occasion to advance that this fact corroborated one of the propositions which he had established in his memoir upon the case of Soufflard—a proposition to the following effect: Bleeding is indicated in the treatment of poisoning from arsenious acid, not only because it acts antiphlogistically, but because it offers the means of abstracting from the current of the circulation a portion of the poison absorbed. This opinion, publicly put forth at the Academy, excited a very lively discussion. The Gazette des Hôpitaux, the doctrines of which were espoused by M. Rognetta, maintained, after a fresh examination of the patient in question, (more than twenty days after the accident,) that he had not suffered any painful palpitations of the heart; that there had been no symptoms of meningitis, but a train of nervous phenomena; that the organic and constitutional action of the arsenic was of an asthenic, or debilitating nature; that bleeding, or other antiphlogistic remedies, acted with the same tendency as the poison itself; finally, that exciting remedies, as the alcoholic preparation, diminished or dissipated the symptoms of intoxication.

There were evidently involved here both a general question belonging to the subject of poisoning with arsenious acid, and another relating specially to the case with which we are occupied. It will be better to devote our attention particularly to the general question; for by basing our arguments upon a solitary fact, much of the interest connected with the toxicological history of arsenious acid, would be lost.

Let us inquire, then, in the first place, if the clinical fact of the cardiac pain and palpitations be well established, or if there be not some error on this subject; in the second place, let us endeavour to estimate the actual value of the presence of arsenic in the blood, (detected at the onset of the poisoning, and a considerable time afterwards.) And let us, finally, consider the question if bleeding acts in the manner supposed by M. Orfila.

Authors who have examined this subject, (Sallin, Desgranges, Renault,) have established from their experiments upon animals, and from the cases which have come under their observation, that there is an excitation of the movements of the heart. M. Cadet de Gassicour, (see Dictionary of the Medical Sciences, article Arsenious Acid,) enumerates among other symptoms of poisoning from arsenious acid, palpitations, followed by great prostration of the strength. None of them speaks of pain of the heart.* M. Alphonse Devergie, (Dictionary of Practical Medicine and Surgery,) says that in these cases, the pulse becomes frequent and more developed, the beating of the heart stronger, &c.; he makes no mention of pain in this region. Laboule and Chaussier observed cases in which the only symptom was repeated syncopes. M. James, in the account of the case of Soufflard, informs us that the pulse was almost imperceptible, and that the beatings of the heart were nearly insensible; the intelligence was not disturbed for a single instant. Lastly, we have seen the results of the experience of MM. Orfila and Coquerel in the last mentioned patient. We should, however, bear in mind that their statement is contradicted by M. Rognetta. Before drawing any conclusion from these premises, it will be well to examine the value of the observations themselves. As often as we apply the ordeal of rigorous criticism to the study of facts, it is rarely that we find them, especially in medicine, undergo it as they should. For, in fact, can we refer the pain existing in the præcordial region invariably to the heart itself? We cannot but doubt this fact, when we call to mind that these theoretical ideas have been admitted only after repeated examinations of the dead body, where the heart has often been found softened, the valves covered with red *plaques*, &c. M. Devergie admits that his experiments upon animals lead him to the conclusion that arsenious acid exercises a special action upon the heart; but, he prudently adds, is this an irritation thrown upon its internal membrane? I say, that we cannot but doubt the constancy of the fact in question, when we bear in mind, on the one hand, that all the alterations described are found in other cases of poisoning, and in other affections, without giving rise to analogous symptoms,—on the other, that pain is an exception to the general rule in M. Bouillaud's cases of endocarditis; and, finally, that this pain may belong to pericarditis, to the cardiac plexus, to the walls of the chest, &c. We may add the statement of M. James, who discovered nothing of the kind in the case of Soufflard, and the denial by M. Rognetta of the assertions of M. Coquerel.

Such, then, is the contradiction which prevails in science upon this point, that we cannot admit it to be a demonstrated fact, that pain in the præcordial region is a pathognomonic sign of poisoning by arsenious acid, since in the last and best observed cases it was wanting; or, in the second place, that this pain belongs specially to the heart, since its seat may be in other neighbouring tissues; or, that the alterations found after death in these cases, are either sufficiently constant, or sufficiently confined to this species of intoxication, to constitute a specific anatomical character of it.

Let us next pass to the subject of the presence of arsenic in the blood. What is its own particular influence, and its effect upon the symptoms which are developed? M. Orfila, to whom unquestionably belongs the very great merit of having pointed out the mode of detecting the presence of this poison in the blood, thinks that it is under the influence of the circulating agent, that the poison, originally absorbed, becomes afterwards spread through the tissues, and determines most, if not all the symptoms which come under observation. This question is certainly one of the most important that can be discussed, not only from its bearing upon the subject of arsenious acid, but upon that of poisoning in general. In fact, we here again encounter the influence of theoretical ideas, and of physiological experiments upon the action of poisons. Every one is acquainted with the investigations of M. Magendie upon the venous capillaries, and the property which he has attributed to them of being the agents of absorption. It should result

* Smith is of opinion that arsenious acid acts upon the heart in a special manner, and that death is the result of the gradual extinction of the contractions of this organ.

from this fact that the blood becomes the most direct cause of all the symptoms consecutive upon the introduction of a poison into the economy. But as we are far from having unanimity in the present day among physicians, this new question of the value of the presence of arsenic in the blood, in cases of poisoning by arsenious acid, necessarily obliges us to enter into some contradictory physiological details.

In 1829, Doctors Thomas Addison and John Morgan, physician and surgeon to Guy's Hospital, published an essay on the operation of venomous agents upon the living body. The object of the labours of these two writers was to determine whether poisons act by passing by absorption into the blood, or whether their effects are produced by simple contact of these agents upon the nervous extremities. It will be at once perceived that the therapeutic consequences which must result from these investigations, are precisely those which are actually interesting us. We cannot find room for an account of all the ingenious experiments performed for the elucidation of this question; we shall content ourselves with the two following:—

1st. Two large bull-dogs, of equal size and strength, were placed upon a table, face to face, and embracing each other; a communication was established between the right carotid of the first and the left carotid of the second. In this manner, the blood starting from the heart of one dog, flowed into the brain of the other. Adopting the theory of venous absorption, it were reasonable to suppose that the dog which furnished blood to his neighbour, would have furnished him also with poisoned blood, if the former should undergo inoculation from any poison, and that then the poisoned blood, carried to the brain, ought necessarily to produce the same effects in both animals. Such, however, were *not* the results observed. Very violent effects manifested themselves in the animal which was giving blood to the other, and which had been poisoned with the extract of nux vomica; and, although the experiment lasted fourteen minutes, during which time the circulation from one to the other was perfectly maintained, not the slightest symptom of the action of the poison was noticed in the other animal.

2d. Between two strong hounds, placed as the preceding, a communication of blood was established through the medium of the jugular veins. Thus, the venous blood from the head of one dog reached the heart of the other. The first was inoculated upon the face with nux vomica, and, shortly after, exhibited all the signs of poisoning with this substance. The experiment lasted seven minutes; the other dog experienced no bad symptom. These experiments were multiplied, and the poison injected directly into the veins or arteries, and, in no instance, did a double poisoning result. From these facts, Messrs. Addison and Morgan concluded that poisons act upon the brain and nervous systems by means of an impression exerted upon the sensitive extremities of the nerves, and not by absorption.

Some of these experiments have been repeated by Dr. F——, for the purpose of verifying these facts. He has ascertained that prussic acid placed upon the foot of a rabbit, after the principal vein of the limb has been carefully tied, kills the animal as quickly as when no ligature has been practised. Another way for the transmission of the poison than the circulatory system must, therefore, be imagined.

Do these experiments demonstrate that the veins have not absorbed the poison, and that it has not passed into the blood? Undoubtedly not: I think that the fact of the presence of the poison in the blood is incontestable. But this is not the question before us; our object is to determine the value of the presence of the poison in the circulatory system, and its real agency in the production of the symptoms. In our view, the experiments which we have cited are of a nature not to explain the proper action of the poison, but to modify the received views of the influence of the circulation in these cases. In fact, the blood appears to contain the poison merely as do the other tissues and fluids of the body; and, to return to our particular subject, the arsenic is found in the blood, just as it is in the muscles, in the bones, and throughout the body; but it is not the cause of the train of symptoms. It is even probable, that long after the disappearance of the symptoms, arsenic may exist in the tissues. M. Orfila, we know, detected it in blood drawn twenty-two days after a poisoning, and when the patient of M. Coquerel was in full convalescence. Further, the arsenic mingled in the blood does not seem to possess the venomous properties elsewhere belonging to it. Thus, according to M. Orfila himself, (Legal Medicine, vol. iii. p. 172, *Arsenious Acid*,) leeches, which expire rapidly in water charged with one-twentieth part of arsenious acid, do not die when they are applied to bodies poisoned by enormous doses of this acid. He has seen them live three days, and did not, at the end of that time, find arsenic in the blood which they disgorged.

What opinion, then, are we to form of the therapeutic action of bleeding, with the object of removing from the current of the circulation a portion of the poison, and of the cause of the symptoms produced? It is evidently a mistaken idea. We are far from rejecting the employment of this remedy; but it must be addressed to other symptoms, and its action be otherwise explained; above all, its indication must be better understood. Bleeding performed for the purpose of diminishing the intensity of the general nervous symptoms, is useless, inasmuch as the poison contained in the blood is not the cause of these symptoms, while they are only increased by the loss of blood. Under a solitary contingency, it may and ought to be practised—when there are evident symptoms of active or passive congestion of one or more organs, important to life. Performed under other circumstances, and without this capital indication, it seems to us, that the tendency of its action will be always in the same direction as that of the poison itself.

